# Diversity of *Fusarium* species isolated from UK forage maize and the population structure of *F. graminearum* from maize and wheat

**DOI:** 10.7717/peerj.2143

**Published:** 2016-06-21

**Authors:** Ryan Basler

**Affiliations:** UMR BIOGER, INRA, Thiverval-Grignon, France

**Keywords:** Trichothecene, *Fusarium graminearum*, Wheat, Genetic relationships, Maize, *F. verticillioides*, *F. culmorum*

## Abstract

Pre-harvest contamination of forage maize by mycotoxin producing *Fusarium* species was investigated in the UK in 2011 and 2012. A total of 15 *Fusarium* species were identified from a collection of 1,761 *Fusarium* isolates recovered from maize stalks and kernels. This study characterized the diversity of *Fusarium* species present in forage maize in the UK. The predominant species detected were *F. graminearum* (32.9%) and *F. culmorum* (34.1%). Along with those species; *F. avenacem, F. cerealis, F. equiseti, F. langsethiae, F. napiforme, F. oxysporum, F. poae, F. proliferatum, F. scripi, F. solani, F. subglutinans, F. tricinctum* and, *F. verticillioides* were occasionally isolated. The trichothecene genotypes for *F. graminearum* were determined to be 84.9% deoxynivalenol (DON) and 15.0% nivalenol (NIV) while *F. culmorum* isolates were determined to have 24.9% DON and 75.1% NIV genotypes. A Bayesian model-based clustering method with nine variable number of tandem repeat markers was used to evaluate the population genetic structure of 277 *F. graminearum* isolates from the maize and wheat in the UK. There were three genetic clusters detected which were DON in maize, NIV in maize and DON in wheat. There were high admixture probabilities for 14.1% of the isolates in the populations. In conclusion, increased maize production in the UK and the high admixture rates in a significant portion of *F. graminearum* populations in maize and wheat will contribute to a new pathogen population which will further complicate breeding strategies for tolerance or resistance to this pathogen in both crops.

## Introduction

Maize is one of the most important crops worldwide, with the cropping area of the 27 member states of the European Union (EU) reaching 9.7 million ha for grain maize and 5.9 million ha for forage maize in 2013. The largest EU maize producers are France, Romania, Germany, Hungary and Italy, which account for 6.1 million ha (FAOSTAT, 2013). With climate change and the establishment of maize varieties tolerant to cooler climates, regions like the UK have seen an increase in maize production. Presently, UK production is at 183 thousand ha (DEFRA, 2014) and is predominantly grown as forage maize on livestock enterprises as a supplement for livestock rations over the winter, with emerging production for bioenergy and grain. The main markets for maize in the EU are for human consumption, animal feed and bioenergy.

Animal pests, weeds and pathogens impact yield and quality of maize (OERKE, 2006) with ear, stalk and roots rots from *Fusarium* species considered to be the most economically significant in the EU (Meissle et al., 2010). The predominant *Fusarium* species associated with ear and stalk rots in the EU are *F. graminearum* followed by *F. verticillioides*, *F. proliferatum* and *F. culmorum* (Meissle et al., 2010). These *Fusarium* species are also capable of producing mycotoxins which contribute to pre-harvest contamination of human food and animal feed impacting health (Desjardins, 2006).

While maize is an emerging crop in the UK, the predominant arable crop is wheat, which accounts for 1.9 million ha (DEFRA, 2014). One of the most devastating diseases of wheat, and small grain cereals, is Fusarium head blight (FHB). The most commonly recovered *Fusarium* species in small grain cereals are *F. graminearum* and *F. culmorum* (Osborne & Stein, 2007). Control of the disease is focused on agricultural practices, fungicides and cultivar resistance. *Fusarium* surveys in cereals in Belgium (Waalwijk et al., 2003) and France (Assemat et al., 1996; Ioos, Belhadj & Menez, 2004) have found that *F. graminearum* is replacing *F. culmorum* as the predominant *Fusarium* species for FHB. Surveys of *Fusarium* species in maize is commonly done in European maize communities with recent studies in Belgium (Scauflaire et al., 2011), Germany (Goertz et al., 2010), Spain (Arino et al., 2007) and Switzerland (Eckard et al., 2011). However, there are presently no surveys being undertaken to determine the *Fusarium* species present in UK maize.

The major mycotoxin type of *F. graminearum* is the trichothecene (TCT) type-B mycotoxin class of fungi capable of producing deoxynivalenol (DON) and its derivatives (3AcDON, 15AcDON) or nivalenol (NIV) (Desjardins, 2006). The NIV-producing isolates of *F. graminearum* have been found to be more aggressive in maize than the DON-producing isolates (Carter et al., 2002). The emergence of NIV-producing populations has been found in regions with maize and wheat production (Pasquali et al., 2010; Sampietro et al., 2011). This has been attributed to maize commonly being rotated with wheat as a part of integrated pest management practices. Typically, maize residues remaining on the soil surface and are present for the following crop. These maize residues may be infested with *F. graminearum* from which ascospores are released to initiate infection of wheat under favorable conditions (Meissle et al., 2010). Research on the biology of *F. graminearum* is needed to understand the infection process and pathogenesis in the life cycle of this pathogen to develop control methods to reduce infection in wheat and other cereals.

Understanding that the natural boundaries and climates of agricultural regions are contributing factors to pathogen potential is an important area to focus research with *Fusarium* species. The UK has a temperate climate with high humidity year round and is separated from Europe by the English Channel and the North Sea. These natural climates and boundaries may select for pathogen profiles that are potentially unique compared to the agricultural regions found in continental Europe. Even with the widely understood effects that *Fusarium* species have on maize production in terms of yield and quality, the knowledge available on the occurrence of these species in UK maize is absent. Additionally, little is known about the population subdivision of *F. graminearum* in the UK based on differences of host species, mycotoxin profiles and pathogen dynamics in wheat and maize.

Therefore, the aim of this study was to determine the *Fusarium* species and the mycotoxin profiles of those species that are naturally occurring in forage maize in geographically diverse fields in the UK. Sampling strategies of maize would focus on identifying predominant mycotoxin *Fusarium* species and emerging *Fusarium* species in UK maize over a two year period. To investigate host specialization and pathogen dynamics of *F. graminearum*, polymorphic allele data was obtained by nine variable tandem repeat markers (VNTR) from 277 isolates of *F. graminearum* recovered from UK maize and wheat, genotyped for TCT-type and analyzed by a Bayesian model-based clustering method and population genetic software.

## Materials and Methods

### Maize sample collection

Sampling of maize plants was undertaken in 2011 and 2012 in the UK to assess the diversity of *Fusarium* species at harvest in five major production regions (North, North Central, South East, South West and West Central) at fifteen fields. The fifteen forage maize fields sampled were trail grounds for maize variety testing which were managed by Limagrain and the British Society of Plant Breeders (Table 1). Sixty-six plants were sampled at each field from the surrounding maize around each trail ground which allowed for the identification of *Fusarium* species present at greater than 5%. Data on the mean daily temperature and precipitation was acquired from the closest weather station to each sampling site from the British Atmospheric Data Centre (National Centre for Atmospheric Science, 2012).

The maize was sampled when dry matter (DM) content was between 28 and 32% as determined by five randomly sampled plants at each field. DM of 28–32% occurs when the maize plant has entered into the reproductive stages of plant development and the kernel begins to turn yellow on the outside while the inner fluid becomes milky white due to accumulating starch. The harvested maize plants were commercial hybrids naturally infected with *Fusarium*. The maize was precision sown in rows with 0.75–0.80 m spacing between rows with an expected density of 100,000 plants per hectare. Sampled plants were selected in a diagonal manner beginning at an arbitrary point at the edge of the field and working towards the center. Each plant was harvested at the base of the stalk, 2–3 cm above the ground, and retained in separate collection bags to avoid cross contamination of samples. Plant samples were stored at 4 °C until they were analyzed for *Fusarium* colonization.

**Table 1:** Sample location of maize samples taken in the UK in 2011 and 2012. Sixty-six plants were harvested at each site when the maize plants had a dry matter content between 28 and 32%.

UK maize region	Sample site	County	Coordinates	Maize cultivar	Year
North	1	Yorkshire^b^	54°19″40′N, 1°27″76′W	NK Smile	2011/2012^c^
North Central	2	Nottinghamshire^a^	53°13″48′N, 0°55″80′W	Artist	2011
	3	Lincolnshire^a^	53°0″79′N, 0°45″02′W	Artist	2011
	4	Shropshire^b^	52°43″06′N, 02°23″46′W	ES Picker	2011/2012^d^
South East	5	Sussex^b^	50°50″55′N, 0°11″33′E	Kokon	2011
	6	Kent^b^	51°15″39′N, 1°05″05′E	Kougar	2012
South West	7	Somerset^a^	51°5″33′N, 2°59″17′W	ES Regain	2011
	8	Devon^b^	50°39″61′N, 3°20″00′W	ES Marco	2011/2012^e^
West Central	9	Oxfordshire^a^	51°56″86′N, 1°12″79′W	MAS17E	2011
	10	Gloucestershire^b^	51°40″35′N, 2°37″47′W	Bonapart	2011
	11	Herefordshire^b^	52°4.74′N, 2°56.31′W	Claxxon	2011/2012^c^

**Notes.**

a Limagrain maize trials.

b BSPB forage maize list trials.

c 150 m North East of 2011 field.

d 100 m North West of 2011 field.

e 100 m East of 2011 field.

### Isolation and identification of *Fusarium* species in maize

*Fusarium* isolates from maize were recovered from kernels, and stalk tissues utilizing a standardized isolation technique. In a laminar flow bench, maize stalk sections were taken in 5 mm sections from either the base of the plant, below the primary ear or above the primary ear for the selected plant. Stalk samples were longitudinally split and tissues of the vascular bundle were removed aseptically from the innermost leaf of the stalk. Three pieces of stalk tissue from the same plant were transferred to 90-mm Petri plates with Spezieller Nährstoffarmer Agar (SNA). Under the same aseptic conditions as the stalk samples, maize kernels were manually removed from the ear and ten kernels were randomly selected for analysis. The maize kernels were surface sterilized in sodium hypochlorite (1% available chlorine) for 2 min and rinsed twice with sterile distilled H_2_O and dried on sterilized paper. After drying completely the maize kernels were transferred to 90-mm Petri plates with SNA at 5 kernels per plate. All samples were incubated at 20 °C under 12 h near-UV and 12 h dark cycle for 3–5 days. Hyphal tips of potential *Fusarium* colonies were transferred aseptically to new 90-mm Petri plates of SNA and incubated under the same conditions for 7–10 days. A conidia and mycelium solution from each sample was preserved in 1.5 ml of 25% glycerol-water and stored at −80 °C. All potential *Fusarium* isolates from the maize samples were retained and identified to species.

*Fusarium* species were initially identified to species based on the morphological characteristics (Leslie & Summerell, 2006) of the macroconidia, microconidia and general mycelium presentation from a single spore isolate grown for 7–10 days on SNA with an Olympus BH-2 light microscope. When macroconidia, microconidia and mycelial characteristics from SNA were insufficient for identifying the species further examination of the samples were done on different agar media. Potato Dextrose Agar (PDA) was used to identify colony pigment characteristics of aerial mycelium on the agar (Leslie & Summerell, 2006). Carnation Leaf Agar (CLA) was used to identify macroconidia, chlamydospores and the presentation of aerial mycelium.

DNA was amplified in multiplex PCR by Goertz et al., (2010) to confirm the identity of ten *Fusarium* species. The ten *Fusarium* species identified in the multiplex PCR are *Fusarium avenaceum*, *F. culmorum*, *F. cerealis, F. equiseti, F. graminearum, F. oxysporum, F. poae, F. proliferatum, F. subglutinans* and *F. verticillioides*. Single spore isolates were grown on PDA plates for 3 days and genomic DNA was extracted from harvested aerial mycelia with a cetyltrimethylammonium bromide (CTAB) method (Leslie & Summerell, 2006). For each amplification reaction 2 µl of template DNA (40 ng/µl) was mixed with 18 µl PCR master mix containing 12 µM of each primer, 6.0 µl of 10× PCR buffer plus 20 mM MgCl2 (Roche, Diagnostics, Mannheim, Germany), 400 µM dNTPs, 0.16 µl of Fast start Taq DNA polymerase (Roche Diagnostics, Mannheim, Germany). The PCR consisted of one cycle of 5 min at 94 °C; 35 cycles at 30 s at 94 °C (denaturing), 30 s at 60 °C (annealing), 1 min at 72 °C (extension); and one cycle of 10 min at 72 °C. After the PCR, 5 µl of the amplification product was used to reveal the presence/absence under UV light on 1.5% agarose gel (Sigma Aldrich, Darmstadt, Germany) containing ethidium bromide (0.5 µg/ml) and scored relative to a 100-bp HyperLadder (Bioline, London, UK).

### Trichothecene genotyping for *F. graminearum* and *F. culmorum*

Multiplex PCR assays were used to characterize the trichothecene genotypes NIV, 3AcDON and 15AcDON based on the polymorphisms in the Tri5 gene cluster of the trichothecene biosynthetic pathway. Targeting more than one *tri* gene was undertaken to reduce misidentification due to gene cluster recombination (Pasquali et al., 2011). The first multiplex assay (Quarta et al., 2006) used primer pairs based on the Tri3 and Tri7 genes. The primers for the Quarta et al. multiplex assay are Tri3F971, Tri3F1325, Tri3R1679, Tri7F340, TriR965 with three DNA fragments for 15AcDON (708 bp), 3AcDON (354 bp) and NIV (625 bp). The second multiplex assay (Ward et al., 2008) used primer pairs based on the Tri12 gene. The primers for the Ward et al. multiplex assay are 12CON, 12NF, 12-15F, 12-3F with three DNA fragments for 15AcDON (670 bp), 3AcDON (410 bp) and NIV (840 bp). Both multiplex PCR reactions for determining the trichothecene chemotype were done in 20 µl reaction volumes with 30 ng DNA as described for *Fusarium* species-specific assays (Goertz et al., 2010). The amplification protocol consisted of one cycle of 5 min at 95 °C; 35 cycles at 30 s at 95 °C (denaturing), 30 s at 55 °C (annealing), 1 min at 72 °C (extension); and one cycle of 10 min at 72 °C. After the PCR, the amplification product was revealed as described for *Fusarium* species-specific assays (Goertz et al., 2010).

### Statistical analysis

The incidence of *Fusarium* species was determined from the predicted mean at each field from a restricted maximum likelihood (REML) analysis to assess the significance of regional locations, chemotypes, and differences between years using Genstat (GenStat, 2011). Multiple strains of the same species recovered from the same plant were assessed qualitatively as their independence could not be critically assessed.

### Sampling and isolation of *F. graminearum* in wheat

For the evaluation of the population structure of *F. graminearum* in maize and wheat the isolates from wheat were recovered from wheat kernel samples which were harvested in 2012 from wheat producers in the UK. A total of thirty-two isolates were recovered from thirty-two fields with one isolate representing each field. The locations of the UK fields are identified in an EU survey of *Fusarium* from 2001 to 2013 (Pasquali et al., 2016). The wheat kernels were screened for *F. graminearum* by surface sterilizing the kernels in sodium hypochlorite (0.37% available chlorine) with 0.1% Tween20 for 5 min and then rinsing twice with sterile distilled water and dried on sterilized paper. 20 wheat kernels per sample were then transferred to 9 cm Petri plates with SNA. Wheat kernel samples were then incubated under the same conditions as maize samples with *F. graminearum* isolates identified as described above.

### Population genetics analysis with variable number tandem repeat markers

Nine VNTR markers (Suga, Gale & Kistler, 2004) were used for the analyses of population genetic structure in the *F. graminearum* populations which were; HK630, HK913, HK917, HK957, HK967, HK977, HK1043, HK1059 and HK1073. There were 245 *F. graminearum* isolates from the maize collection and 32 isolates from wheat collection used for the population genetics analysis. VNTR amplifications were performed with 20 ng of DNA per reaction in 10 µl of master mix as described in the *Fusarium* species-specific assays. PCR was performed as described by Ward et al. (2008) which consisted of 2 min at 95°C; 25 cycles of 1 min at 95 °C, 1 min at 59 °C and 1 min at 72 °C with a final extension stage of 10 min at 72 °C. Reaction products were scored relative to GS500 ROX (Applied Biosystems) internal size standard using an ABI 3730xl Genetic analyzer with Genemapper 4.0 software. Irresolvable alleles and missing data were treated as missing data.

Genetic diversity and *F*_ST_ based genetic distances (Slatkin, 1995) were estimated with ARELQUIN 3.5 (Excoffier, Laval & Schneider, 2005). Pairwise *F*_ST_ distance estimates were determined based on the number of different alleles and the significance of pairwise *F*_ST_ estimates using 1,000 permutations. The analysis of the admixture was performed using Bayesian method with the software STRUCTURE 2.3.4 (Pritchard, Stephens & Donnelly, 2000). The estimation of the number of populations was calculated based on the second order rate of change of the likelihood (Evanno, Regnaut & Goudet, 2005).

## Results

### Incidence study in maize

The two year maize sampling resulted in a collection of 1,761 *Fusarium* isolates which were identified to species from the sampling of 2,970 maize stalk pieces (967 *Fusarium* isolates) and 9,900 maize kernels (794 *Fusarium* isolates). There were only three ears and one stalk sample that had observable *Fusarium* mycelium out of all of the samples evaluated. This study identified fifteen *Fusarium* species in maize with the predominant species being: *F. graminearum* (32.9%), *F. culmorum* (34.1%), *F. solani* (19.8%), *F. cerealis* (3.5%), *F. avenaceum* (3.0%) and *F. poae* (2.8%). Other *Fusarium* species identified in the collection were: *F. oxysporum* (1.2%), *F. verticillioides* (1.1%), *F. proliferatum* (0.4%), *F. tricinctum* (0.3%), *F. subglutinans* (0.3%), *F. langsethiae* (0.2%), *F. napiforme* (0.2%), *F. equiseti* (0.2%) and one isolate of *F. scripi* (Table 2). The species-specific assay for * F. avenaceum* (Turner et al., 1998) may not differentiate between *F. avenaceum* and the closely related *F. tricinctum* (Kulik, 2008; Tan & Niessen, 2003). Differentiation of *F. avenaceum* and *F. tricinctum* was not always conclusive with macroconidia, therefore we confirmed the species based on differences in the formation of microconidia.

**Table 2:** Percentage incidence of *Fusarium* species recovered from UK maize in 2011 and 2012.

UK maize region	Sample site	Year	*F. avenaceum*	*F. cerealis*	*F. culmorum*	*F. equiseti*	*F. graminearum*	*F. langsethiae*	*F. napiforme*	*F. oxysporum*	*F. poae*	*F. proliferatum*	*F. solani*	*F. subglutinans*	*F. tricinctum*	*F. verticillioides*
North	1	2011^K^	1.5	1.5	6.1	–	15.2	–	1.5	1.5	6.1	–	15.2	–	–	–
		2011^S^	9.1	1.5	22.7	–	66.7	–	–	–	–	–	22.7	–	–	–
		2012^K^	–	–	–	–	4.5	–	–	–	–	–	15.2	–	–	–
		2012^S^	3.0	7.6	34.8	–	27.3	–	–	–	–	–	24.2	–	–	–
North Central	2	2011^K^	6.1	1.5	39.4	–	48.5	–	–	–	6.1	–	19.7	–	–	–
		2011^S^	6.1	–	39.4	–	16.7	–	–	1.5	–	–	15.2	–	–	–
	3	2011^K^	7.6	1.5	16.7	–	34.8	–	1.5	–	1.5	–	6.1	–	–	–
		2011^S^	6.1	3.0	25.8	–	24.2	–	–	1.5	–	–	24.2	–	1.5	1.5
	4	2011^K^	–	16.7	51.5	–	6.1	–	–	–	–	–	3.0	1.5	–	—
		2011^S^	–	6.1	54.5	–	4.5	–	–	1.5	–	–	9.1	–	–	–
		2012^K^	–	–	7.6	–	6.1	–	–	–	–	–	45.5	–	–	–
		2012^S^	–	1.5	34.8	–	19.7	–	–	–	–	–	54.5	–	–	–
South East	5	2011^K^	–	1.5	18.2	–	28.8	–	–	–	1.5	–	9.1	–	–	–
		2011^S^	1.5	3.0	28.8	1.5	48.5	–	–	–	3.0	–	7.6	–	–	–
	6	2012^K^	–	–	–	–	6.1	–	–	–	–	–	6.1	–	–	–
		2012^S^	–	–	10.6	–	37.9	–	–	–	–	–	36.4	–	–	–
South West	7	2011^K^	3.0	3.0	13.6	–	10.6	1.5	–	–	9.1	1.5	6.1	–	–	–
		2011^S^	–	1.5	27.3	–	22.7	–	–	–	–	–	12.1	–	–	1.5
	8	2011^K^	1.5	1.5	37.9	–	33.3	–	–	–	3.0	–	9.1	–	–	–
		2011^S^	1.5	3.0	31.8	–	39.4	–	–	–	–	–	7.6	–	–	–
		2012^K^	–	–	–	–	–	–	–	–	–	–	–	–	–	–
		2012^S^	–	3.0	9.1	–	25.8	–	–	–	–	1.5	43.9	–	–	–
West Central	9	2011^K^	–	1.5	10.6	–	54.5	–	–	1.5	13.6	3.0	18.2	–	–	–
		2011^S^	3.0	4.5	28.8	–	47.0	1.5	–	–	–	–	10.6	–	–	–
	10	2011^K^	–	6.1	10.6	1.5	18.2	–	–	–	3.0	1.5	4.5	–	–	–
		2011^S^	1.5	4.5	16.7	0.0	33.3	–	–	–	–	1.5	1.5	–	–	–
	11	2011^K^	4.5	4.5	31.8	–	6.1	1.5	1.5	1.5	4.5	–	9.1	–	–	1.5
		2011^S^	10.6	1.5	45.5	–	3.0	–	–	1.5	–	–	19.7	–	1.5	–
		2012^K^	1.5	1.5	3.0	1.5	–	–	–	–	–	–	7.6	–	–	–
		2012^S^	4.5	–	31.8	–	30.3	–	–	–	–	–	27.3	–	–	–
	Total 2011^K^		2.4	3.9	23.6	0.2	25.6	0.3	0.5	0.5	4.8	0.6	10.0	0.2	0.2	0.2
	Total 2011^S^		3.9	2.9	32.1	0.2	30.6	0.2	–	0.6	0.3	0.2	13.0	–	0.9	0.3
	Total 2012^K^		0.3	0.3	2.1	0.3	3.3	–	–	–	–	–	14.8	–	–	–
	Total 2012^S^		1.5	2.4	24.2	–	28.2	–	–	–	–	0.3	37.3	–	–	–

**Notes.**

K Kernel tissue S Stalk tissue – no *Fusarium* spp

^1^ Yorkshire; ^2^ Nottinghamshire; ^3^ Yorkshire; ^4^ Shropshire; ^5^ Sussex; ^6^ Kent; ^7^ Somerset; ^8^ Devon; ^9^ Oxfordshire; ^10^ Herefordshire; ^11^ Gloucestershire.

**Figure 1:**
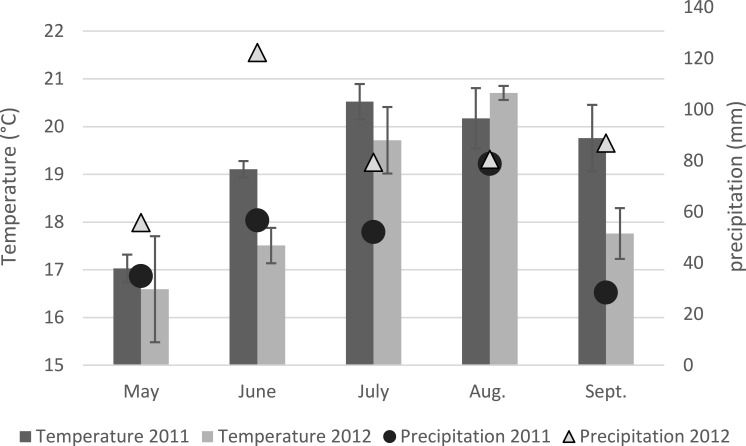
Mean maximum temperatures and precipitation on the four fields sampled in the UK in 2011 and 2012.

Across all fields sampled in the UK, the incidence of *Fusarium* colonized maize plants with at least one *Fusarium* isolate recovered in either stalk of kernels was 85% ± 2.7 SD for 2011 and was 86% ± 0.08 SD for 2012. *F. solani* regularly recovered as a saprophyte was found to have co-occurred with another *Fusarium* species for 85% ± 0.18 SD of the samples for both years. Of the 990 maize plants sampled, *F. graminearum* (44.0% ± 0.14 SD) or * F. culmorum* (31.0% ± 0.16 SD) were present in 63.9% ± 0.12 SD of the plants. The incidence of *F. graminearum* and *F. culmorum* between fields was insignificant within the same year. The incidence per field of *F. graminearum* was significantly (*P* = 0.002) greater in 2011 than in 2012 as was the incidence of both *F. graminearum* and/or *F. culmorum* (*P* < 0.001). The incidence per field by *F. culmorum* was not significantly (*P* = 0.139) different between 2011 and 2012. The mean maximum temperatures and mean precipitation for the sites in Yorkshire, Shropshire, Devon and Gloucestershire are shown in Fig. 1 for the growing seasons in 2011 and 2012.

### Trichothecenes for *F. graminearum* and *F. culmorum*

Trichothecene types for *F. graminearum* and *F. culmorum* isolates in maize had consistent results for all isolates in both multiplex PCR assays. The *F. culmorum* and the * F. graminearum* isolates from maize chemotypes of DON (3AcDON-type and 15AcDON-type) and NIV-type are shown in Table 3. The *F. graminearum* isolates were predominantly the 15AcDON-type (84.1%) followed by NIV-type (15.0%) and three isolates of 3AcDON-type. The *F. culmorum* isolates were predominantly the NIV-type (75.1%) followed by 3AcDON-type (24.0%) and three isolates of 15AcDON-type. The *F. graminearum* isolates from wheat used in the population genetic study were all determined to be 15AcDON-type.

### Population structure and diversity of *F. graminearum* populations

**Figure 2:**
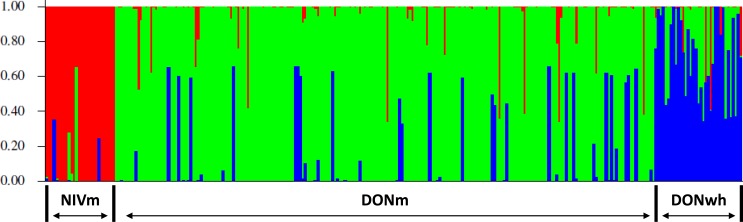
Representation of admixture estimates based on VNTR data for *F. graminearum* isolates from UK wheat and maize. Red, NIV maize; green, 15AcDON maize; blue, 15AcDON wheat.

Three genetic clusters (*K* = 3) was determined to capture the major genetic structure in our data set based on the highest $\mrm{\Delta }K$ (<10). The three clusters were determined from the evaluation of the genetic variation based on the VNTR loci utilizing a Bayesian clustering method for admixture in the 245 (217 DON-type and 28 NIV-type) *F. graminearum* isolates from maize and the 32 DON-type isolates from wheat. The clusters corresponded and were organized based on the host species and the TCT-type of the isolates. The three groups were NIV-type from maize (NIVm), 15AcDON-type from maize (DONm) and 15AcDON-type from wheat (DONwh). The admixture estimates for each isolate is shown in Fig. 2. The pairwise *F*_ST_ distance estimates indicated significant differences between the groups based on trichothecene chemotype; NIVm and DONm (*F*_ST_ =0.2207, *P* < 0.001) and NIVm and DONwh (*F*_ST_ =0.2038, *P* < 0.001). There were also differences between host species with the same trichothecene chemotype; DONm and DONwh (*F*_ST_ =0.033, *P* < 0.05).

**Table 3:** Geographical origin, year, mean number of isolates per site, species and trichothecene genotype of *Fusarium* species from maize.

Maize growing area	Year	Mean isolates/field	*F. culmorum*			Mean isolates/field	*F. graminearum*		
			3AcDON	15AcDON	NIV		3AcDON	15AcDON	NIV
North	2011^a^	19	8	0	11	48	0	47	1
	2012^a^	23	0	0	23	21	0	20	1
North Central	2011^c^	50.7	11.7	0.3	38.7	28.7	0.3	24.7	3.3
	2012^a^	28	6	0	22	16	1	16	0
South East	2011^a^	32	9	0	23	42	1	37	4
	2012^a^	7	1	0	6	29	0	26	3
South West	2011^b^	36	15.5	0	20.5	33.5	0	14	19.5
	2012^a^	6	0	0	6	17	0	17	0
West Central	2011^c^	31.7	6	0.7	25	35.3	0	32.3	3
	2012^a^	25	3	0	22	21	0	19	2
All regions	2011^d^	37	10.1	0.3	26.6	34.8	0.2	28.3	6.3
	2012^d^	17.8	2	0	15.8	21	0.2	19.6	1.2

**Notes.**

a One field sampled.

b Two fields sampled.

c Three fields sampled.

d Mean all fields for year.

3AcDON 3,acetyldeoxynivalenol 15AcDON 15,acetyldeoxynivalenol NIV Nivalenol

**Figure 3:**
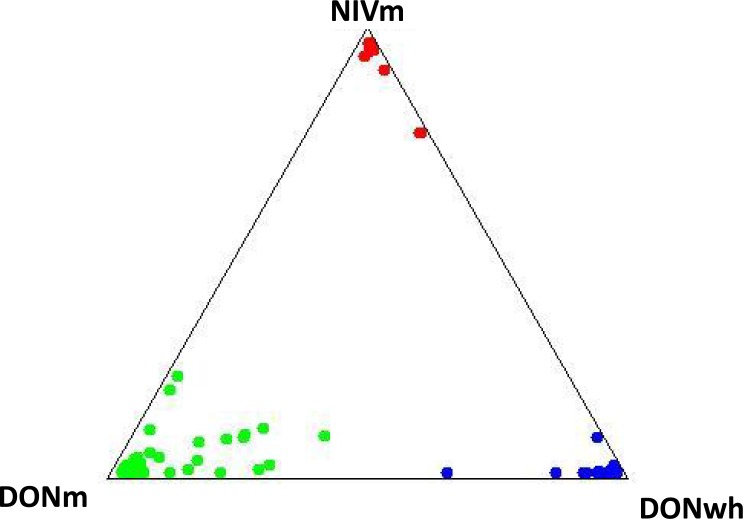
Isolate distribution for *F. graminearum* isolates. Red, NIV maize; green, 15AcDON maize; blue, 15AcDON wheat. Does not include isolates with admixture probabilities greater than 95%.

Admixture probabilities greater than 95% were obtained for 39 of the 277 isolates analyzed. Bayesian posterior mean estimates for the proportion of the genome for the DONm isolates that is derived from NIVm ranged from 13 to 98%. DONwh isolates had a significant (*P* < 0.05) admixture with genetic material shared with DONm isolates. The DONm isolates with high admixture shared a significant (*P* < 0.05) amount of genetic material with NIVm isolates. A visual representation of isolate distribution based on VNTR data for the *F. graminearum* populations minus the isolates with admixture probabilities greater than 95% is shown in Fig. 3.

## Discussion

*Fusarium* is a major pathogen of maize and wheat responsible for severe crop loss and mycotoxin contaminations (Charmley et al., 1995; Logrieco et al., 2003). In the present study, we identified the biodiversity of *Fusarium* species from maize in the UK. An extensive collection of 1,761 isolates identified 15 species and characterized the mycotoxin profiles of the predominant species of *F. graminearum* and *F. culmorum*. The prevalent TCT-types of the predominant species were 15AcDON-type and NIV-type for *F. graminearum* and 3AcDON-type and NIV-type for *F. culmorum*. We also identified unique isolates of *F. culmorum* with the 15AcDON-type, *F. graminearum* with the 3AcDON-type and *F. verticillioides*. *F. verticillioides* is a species that produces the mycotoxin T2 and is typically found in warmer maize growing regions in Europe. Additionally, we determined three subpopulations of *F. graminearum* isolates from maize and wheat based on trichothecene profile and host from population genetic analysis approaches. We believe that this study provides comprehensive information on important *Fusarium* species and their associated mycotoxins for maize and wheat grown in the UK.

Environmental factors and host species have a strong impact on the occurrence of a specific chemotype and the incidence of *Fusarium* species. The distribution of *Fusarium* species in maize is influenced by optimal climatic conditions, pathogenicity and competition between other fungi (Doohan, Brennan & Cooke, 2003). Year-to-year variability is one type of environmental factor identified in the incidence of *Fusarium* species as demonstrated in recent EU maize surveys (Dorn et al., 2009; Goertz et al., 2008; Scauflaire et al., 2011). In those studies the prevalence of species varied year-to-year and was believed to be associated with the differences in climatic conditions between years. In this study the warm and drier conditions in June, July and September in 2011 compared to the same fields in 2012 may have contributed to year-to-year incidence differences for *F. graminearum* and other *Fusarium* species in those fields. The growing conditions in 2011 were characterized by dry conditions in the early stages of maize development with moderate precipitation and warm weather in the later stages of plant development until anthesis. In 2012, moderate temperatures and precipitation was present in early stages of maize development with heavy precipitation and cooler temperatures in later stages of plant development. As was reported by Doohan, Brennan & Cooke (2003), *F. graminearum* is more prevalent when at anthesis there is warm weather with moderate precipitation as was present in the 2011 sampling. Reis (1990) determined that the dispersal of ascospores is halted in heavy rain as was present in 2012 and could have been an additional factor for the reduced presence of *F. graminearum* in that year compared to the previous year. The variation in climatic conditions present in the two years of the study probably contributed to the year-to-year differences in the incidence of *F. graminearum* and could also be extended to the other *Fusarium* species and strain types identified in this study.

In maize, wheat and barley in European countries the dominant FHB species has been identified as *F. graminearum* (Eckard et al., 2011; Goertz et al., 2010; Prodi et al., 2009; Talas, Parzies & Miedaner, 2011). An evaluation of Italian *Fusarium* species indicates that climatic conditions influence the prevalence of 15AcDON-type and 3AcDON-type strains (Covarelli et al., 2015). The 15AcDON-type is believed to be prevalent in regions with cool winter climates (Prodi et al., 2009) such as the UK, which is supported in a previous study of UK chemotypes of *F. graminearum* (Jennings et al., 2004). The prevalence of 15AcDON-types of *F. graminearum* from maize and wheat in this study are further support of this chemotype for this climate. In Norway, the 3AcDON-type of *F. graminearum* was identified as the predominant chemotype (Aamot et al., 2015). In North America the increased presence of 3AcDON-type since 1998 is suggested to be from shifts in agricultural practices or environmental conditions (Ward et al., 2008). The presence of the 3AcDON-type in the UK may be attributed to similar changes in the landscape with isolates originating from Norway where this is the predominant chemotype (Aamot et al., 2015). In Europe the DON chemotype of the *F. culmorum* population (Pasquali et al., 2016) has been exclusively identified as the 3AcDON-type but the species is known to produce 15AcDON (Mirocha et al., 1994). The presence of the 15AcDON-type isolates of *F. culmorum* in this study may be a subpopulation in Europe not yet identified but would require further evidence in additional studies to confirm this subpopulation. The sampling method used for this study was intended to capture the presence of species present at greater than 5% in each field which would also capture novel characteristics of chemotypes of subpopulations. New emergence, climate change, sampling method and host species could be contributing factors for the identification of the 3AcDON-type of *F. graminearum* isolates and 15AcDON-type of *F. culmorum* isolates found in this study.

The oxygenation or acetylation of TCT, including 15AcDON and NIV, alters the toxicity and bioactivity (Kimura et al., 1998) and may affect the frequency of a specific TCT-type. The 15AcDON-type and 3AcDON-type chemotypes are acetylated derivatives of DON (Miller et al., 1991) while NIV is an oxygenated C-4 (Kimura et al., 2007). Modification of the bioactivity occurs from the structural differences of oxygenation and acetylation which may affect strain fitness (Ward et al., 2002). Studies in terms of fecundity, growth rates and toxicity have identified the 3AcDON-type isolates as the dominant population type over the NIV-type (Zhang et al., 2012) and 15AcDON-type (Ward et al., 2008). However, the NIV-type is believed to be a virulence factor in maize (Carter et al., 2002; Maier et al., 2006) and is not affected by climatic conditions (Covarelli et al., 2015). The presence of the NIV-type *F. graminearum* and *F. culmorum* isolates found in this maize sampling may be an indicator of the virulence factor of NIV producing strains in maize. However, additional study of this potential of NIV being a virulence factor would require greater depth of investigation than addressed in this study.

The association of chemotypes with distinct *F. graminearum* populations has been found in wheat (Karugia et al., 2009; Ward et al., 2008), barley (Burlakoti et al., 2011) and maize (Qiu & Shi, 2014). While this study evaluated the population genetics of *F. graminearum* isolates from a single year, multiple year population studies identified shifts in isolates based on chemotypes (Guo, Fernando & Seow-Brock, 2008; Karugia et al., 2009; Ward et al., 2008). Population genetic studies of *F. graminearum* in wheat have indicated that the direction of gene flow was going from 3AcDON-type towards NIV-type (Zhang et al., 2012). In this study of population genetics of *F. graminearum* there were NIV-type and 15AcDON-type isolates of *F. graminearum* from maize and 15AcDON-type isolates from wheat. Isolates were represented by a VNTR haplotype and assigned to a cluster based on chemotype and host species of each isolate. The three clusters were identified as: maize 15AcDON-type, maize NIV-type and wheat 15AcDON-type. Fourteen percent of the isolates demonstrated strong admixture probabilities with other genetic clusters than their assigned cluster. The wheat DON-types with high admixture shared significant genetic material with the maize DON-types while the maize DON-types with high admixture shared significant genetic material with the maize NIV-types. The prevalence of sexual reproduction of field populations of *F. graminearum* populations and spore dispersal may explain the gene flow between chemotypes identified in other studies (Guo, Fernando & Seow-Brock, 2008; Karugia et al., 2009; Ward et al., 2008) and explain the high admixtures in this study.

*F. graminearum* is an important etiological agent on wheat and maize. The sexual ascospores and asexual macroconidia of *F. graminearum* overwinters in maize crop residues with dispersal favored by moist warm conditions (Schmale, Arntsen & Bergstrom, 2005; Paul et al., 2004). While subpopulations of the species are restricted by natural barriers, which includes geographical distances, sea and mountains, Karugia et al., (2009) studies on the long-distance dispersal of ascospores (Maldonado-Ramirez et al., 2005) have found that ascospores are present at high altitudes which would overcome those natural barriers. The high precipitation in the UK, the increase in maize production and the high admixture rates between *F. graminearum* populations in maize and wheat presents an opportunity to introduce a pathogen population that may be more toxigenic and more vigorous which would have significant implications for food safety in the UK.

In conclusion, this study provides the important first description of *Fusarium* species in UK maize. The identification of the predominant *Fusarium* species, *F. graminearum* and * F. culmorum*, along with potentially emerging species (*F. verticillioides*) and subpopulations of species (3AcDON *F. graminearum*; 15AcDON *F. culmorum*) in UK maize will enable maize breeding programs to focus their resistance strategies on these pathogens. The identification of subpopulations within *F. graminearum* associated with their host species and chemotype profile provides an important informative baseline for future epidemiological surveillances. Given that wheat is a significantly greater economical crop compared to maize in the UK, continued monitoring and evaluation of the TCT-type populations of *F. graminearum* population in both crops will be of great benefit for breeders and growers. We believe that future epidemiological surveys should continue to monitor the NIV-type populations of *F. graminearum* in both maize and wheat.

##  Supplemental Information

10.7717/peerj.2143/supp-1 Data S1 Data sets for the *Fusarium* species diverstity in maize; mycotoxin profiles for *F. graminearum* and *F. culmorum*; VNTR data for *F. graminearum*

## Additional Information and Declarations

### Data Availability

The following information was supplied regarding data availability: The raw data has been supplied as Data S1.
